# On the Use and Abuse of Purgative Medicine

**Published:** 1825-03

**Authors:** 


### Art. V.—*On the Use and Abuse of Purgative Medicine.* By Dr. Kinglake.

There is no department of medical practice that is more important to the welfare of mankind than the correct administration of purgative medicine. Momentous as are its objects, and fraught as it is with the most serious consequences, it is resorted to with a familiarity and confidence implying that its operation, however powerful, is still harmless. This opinion arises from a misapprehension that the peristaltic or contractile power of the intestines may be indefinitely excited without incurring any considerable ailment,—that the evacuant effect affords relief,—and that no material inconvenience is likely to result from the disordered action that is induced. This is an erroneous notion, and does not sufficiently discriminate the condition of intestinal function that may or may not need the aid of purgative excitement.

The healthy function of the intestinal canal requires that its contractile power should be duly active, at once to preserve its natural energy and to obviate accumulations that would prove deleterious by bulk as well as quality. The peristaltic power of the intestines is essentially connected with the digestive process, as commenced in the stomach and proceeding to its ultimate completion in the bowels. If deficiencies occur in this function, redundant and ill-conditioned feculencies are the necessary consequence, which will produce hurtful distention and irritation.

If this state be protracted and daily aggravated by new accessions of offensive accumulations, it is easy to perceive that a source of disease must be created that will molest and disorder in various degrees and forms of violence. This morbid state is too constantly generated by obtruding on the stomach indigestible quantities of alimentary and other substances, which reaching the bowels in a noxiously crude state, oppress and distend them, so as to render the contractile power incapable of transmitting such an unwieldy mass of obstructing contents. An early consequence of this *remora*, or unseasonable detention of undigested materials, is a state of intestinal distention that incapacitates the contractile power for spontaneous exertion, similar to what occurs in an over-distended urinary bladder, and in the vascular plenitude that is characterised by the morbid condition of arterial and venous plethora. The capability of contracting in those cases is suspended by the existing fulness, which acts as a dead weight on the vital power of the distended parts, sinking it into a state of torpor or deficient action, and sympathetically extending, either to particular parts or to the system at large, morbid disturbances of greater or less severity, according to constitutional and other attending circumstances.

The indication for the use of purgative medicine is confined to the necessity for removing substances from the intestines, that have been unhealthily amassed, owing to an inefficient action of the peristaltic power. A depression of vital energy in the transmitting exertion of the bowels, with the augmenting redundancy of noxious contents arising from such an enfeebled state, is a condition of disease of insidious growth and portentous tendency. It occurs much more frequently than is apprehended, and perhaps may be truly said either to occasion or aggravate a great majority of the morbid derangements that disturb the healthy action of vital power. Indigestion with its endless train of distempered feelings, as well in the first passages as in remote parts of the frame, the various forms of febrile excitement and nervous agitations that disorder the healthful state, may be justly referred to the pernicious influence of inactive and surcharged bowels.

This unnatural state imperiously requires efficient relief, and affords an ample warranty for the free and unrestricted use of purgative medicine, until it be fully corrected and overcome. To desist before this has been accomplished, is to fail in rendering the necessary assistance. The purgative means, therefore, should be continued until the alvine discharges shall have been sufficiently copious to have dislodged all that could have offended, and to have enabled the peristaltic or contractile power of the intestines to resume and steadily perform its healthy action.

This extent of requisite aid will be evinced by the displacement of ill-conditioned feculencies, and the restoration of such as exhibit the appearance of being the indecomposible remains of alimentary substances, with such bilious, pancreatic, and mucous admixtures as are naturally deposited in the intestinal canal for seasonable expulsion. If these excrementitious substances be unduly retained, a source is laid for immediate inconvenience, increasing disease, and eventually for distempers that would appear to have been induced by other causes, and which, unhappily for appropriate and effectual treatment, are subjected to modes of remedy not less inapplicable than unavailing.

Whatever may be the existing debility, however the general strength may be either depressed or exhausted by protracted ailment, if obstructed, or rather scantily relieved bowels be the lurking cause, no adequate remedy can be obtained without exciting the prevailing torpor in a manner that would renew an healthy exertion of the peristaltic power, and thereby remove noxious redundancies and prevent a re-accumulation of them.

The usual remedies of bleeding, vomiting, sudorific, and alterative medicine, directed on these occasions to relieve active symptoms, to determine to the surface, and to correct and improve vitiated secretions, may respectively alleviate, but cannot afford the requisite aid. Whilst the bowels remain inactive on their unwieldy contents, incessantly becoming larger in quantity and worse in quality, no measure of relief short of the direct and sufficient removal of this offending cause can furnish the necessary assistance. All other remedies but those which act as evacuants in the first passages are subordinate and of minor consideration. An excitement of the peristaltic power of the intestines unremittedly sustained until the evil of redundant and vitiated secretions be removed, is the sole remedy that merits reliance, or that is capable of fulfilling the curative indication which existing circumstances furnish.

After this object has been effected, the general strength instead of having been reduced by the purgative process, will have been sensibly amended, and every morbid feeling that has been generated by the prevailing inaction of the bowels, whether near the seat of disease or more remote, whether in the form of local pain or of general irritation, will more or less speedily subside, and satisfactorily evince that the correct mode of treatment has been adopted. When this beneficial result has been obtained, it would be injurious to persist in the use of purgative medicine. The object for which it was instituted having been accomplished, it would be superfluous, harassing, and perhaps destructively injurious, indefinitely to push its influence. To go beyond the point that is indicated by the circumstances of the case, is to act without authority and to incur a greater or less degree of positive mischief. The advantages of necessary and legitimate purgation are inestimably great; but the injurious consequences of carrying the remedy too far may be deplorably severe, and even irretrievably disastrous.

The scybalous and indurated collections that are apt to amass in the intestines, are often large in quantity, and are probably sometimes partially retained for many weeks, instead of being daily evacuated. When thus impacted and confined, the stimulating influence of brisk cathartic medicine is necessary to dislodge the obstruction. The more mild or slightly laxative kind of medicine may induce scanty discharges, or the escape of the more liquid parts of the excrementitious contents between the villous coat of the bowels and the obstructing substances. Castor-oil, manna, the neutral salts, and such lenient articles, will pass over the impeding obstacles to a free discharge, without effecting the necessary relief. Calomel, jalap, scammony, aloes, &c. given in active doses at intervals of six hours, until the requisite expulsion has been effected, and the subsequent evacuations have become well-conditioned, will be found alone commensurate with the benefit that should be procured. Not only the local inconveniences in the region of the abdomen, but every feeling of a febrile and nervous character, the chilliness that indicates an unequal distribution of the circulating fluids, and the congestive fulness on visceral parts denoting local plethora, will subside when the intestinal canal has been sufficiently disencumbered, and its contractile power has been restored to a firm and natural state of transmitting or evacuating action.

Indolent and oppressed bowels soon induce a derangement of the biliary secretion, rendering it either excessive, deficient, or vitiated. The gastric, pancreatic, and even mucous secretions from the villous coat of the intestines, are affected by an unhealthy state of the peristaltic action. This morbid condition of the bowels, shown in an irregular and insufficient exertion of their contractile power, obtains slowly, and gradually establishes itself, so as to assume a character of disease that is too obscure to be readily recognised, but which possesses a latent severity that undermines health at its chief source, that of nutritive digestion.

If the bowels perform not their allotted portion of the digestive process, and fail in forwarding by seasonable expulsion the feculent remains of alimentary substances, deficient nutrition and morbid excitement must inevitably ensue. It is the object of purgative medicine fully to discharge all lingering redundancies from the intestinal canal, that the distempered action of the first passages may be overcome, and the various secretions naturally deposited in them be duly restored, together with the healthful tone and activity of the peristaltic power. Those advantages being effected, the diseased sympathies that may have been awakened in different parts of the frame will cease, and the salutary action of vital power will resume its wonted establishment.

An unerring test of those benefits having been obtained, is afforded by a renewal of the natural state of the intestinal evacuations, both in respect of quantity and quality. Great and blameable remissness occurs in medical practice, in not actually inspecting the condition of those discharges, when the bowels have been irregular and defective in their transmitting function. The reports of unprofessional persons on this subject, are too vague and undiscriminating to be at all satisfactory. The importance of the object merits the closest attention, and should never be neglected as unworthy of notice. It is by the colour, consistence, odour, and the greater or less uniformity in the substance of the intestinal excrements, that either the degeneracy from the natural state is to be estimated, or the restoration to the healthy condition may be inferred. The bulk as well as aspect of those evacuations should be regarded. Scanty discharges will speedily vitiate the quality, and when the appearance is unhealthy, it is fair to conclude that the evacuations are insufficient. True it is therefore, that the quantity and quality of the contents of the bowels are correlative conditions, mutually involving each other's existence.

A renovation of the healthy state of the intestinal canal, in reference to its digestive and transmitting function, is the curative intention of purgative medicine in those morbid cases that have arisen from constipated bowels and a faulty state of the alvine secretions. When this correction of disease has been effected, no farther warranty remains for prosecuting a cathartic mode of treatment. If purgative medicine be urged beyond this point, it will exceed the salutary limit, and produce rather than remove disease.

When oppressive distention of the bowels, and the consequent torpor or deficient energy occasioned by it, have been sufficiently relieved, it would be to risk incurring a positive mischief to persist in the use of purgative medicine. This error, however, is not unfrequently committed, and the deplorable consequence is an increased debility of the intestinal canal, with an additionally vitiated state of the various secretions entering into its cavity. When a ready transit of bile is afforded from the liver to the duodenum, and the abdomen is free from hardness or distention, as the result of purgative medicine, the reasonable presumption is, that the purpose meant to be served has been fully answered; and to push evacuation further, would be to substitute ulterior disease for the relief which had been obtained.

It is observable that an unnecessarily protracted use of purgative medicine is apt to induce the aspect of vitiated discharges which the early employ of them removed; and were this ideally morbid appearance to be assumed as an authority for the continuance of the mistaken remedy, the most injurious effects may be produced, among which may be enumerated, a detachment of the mucus necessary to defend the villous coat of the intestines from the stimulating acrimony occurring in the first passages; or the long-continued excitement may so stimulate the secerning vessels supplying the mucous covering, as to cause them to furnish a fluid too thin and irritating to serve that defensive purpose, or even to produce blood itself, shown in sanguineous or dysenteric evacuations. These occurrences appertain to the evils of mal-treatment, and for the production of which a correct application of the practice cannot be held amenable.

If general excitement, visceral inflammation, or any kind of functional disturbance, whether sensorial or irritative, should be effectively remedied by appropriate treatment, it would be preposterous to persist in the means of relief that had been successfully employed. Such work of supererogation would be necessarily pernicious, and likely to induce a state of ailment not less perilous than that which a correct use of the remedial means would have permanently removed. In the employ of purgative medicine, a similar limit should be observed. The intestines, when oppressed and unfitted for a due performance of their function, require to be evacuated to a given extent; beyond which, the relief sought for would not be obtained, but, on the contrary, disease would be the probable result.

Remedies are allied to certain morbid states needing their salutary agency. When those states have been sufficiently corrected, a continuance of the agency would be to direct its influence against the relieved or healthy condition, which, if efficiently operative, must produce more or less of malady. To transmute remedial into noxious agents by an unlimited or licentious use of their powers, is to invert the order of medicinal aid, by causing it to generate rather than subdue disease.

*Taunton; January 9th,* 1825.

